# Role of Oxidative Stress and Neuroinflammation in the Etiology of Alzheimer’s Disease: Therapeutic Options

**DOI:** 10.3390/antiox14070769

**Published:** 2025-06-23

**Authors:** Marta Weinstock

**Affiliations:** Institute for Drug Research, Faculty of Medicine, The Hebrew University, Jerusalem 9112001, Israel; martar@ekmd.huji.ac.il

**Keywords:** synaptic plasticity, mitochondrial dysfunction, oxidative stress, microglial reactivity, pro-inflammatory cytokines, antioxidant drugs, anti-inflammatory drugs

## Abstract

Cognitive impairment in subjects with Alzheimer’s disease correlates well with the loss of synaptic plasticity. This results from mitochondrial dysfunction and production of reactive oxygen species, which damage nerve terminals causing them to release ATP and adenosine. These purines activate receptors on microglia resulting in a change in morphology and release proinflammatory cytokines that exacerbate neuronal damage. The review describes retrospective studies with naturally occurring antioxidants, vitamin E, resveratrol, Ginkgo biloba and others that suggested they reduce the incidence of Alzheimer’s disease. They have antioxidant activity in cellular systems and rodent models, but most of them failed in clinical trials, probably because they were not absorbed after oral administration or, like anti-inflammatory drugs, were not given at the right time or for long enough to detect an effect on disease progression. Ladostigil is an aminoindan derivative that is well absorbed after oral administration. It has antioxidant effects in cells and prevents cytokine release from activated microglia. In a phase 2 trial in subjects with mild cognitive impairment, ladostigil significantly reduced number of converters to Alzheimer’s disease in ApoE4-ve subjects and delayed the decline in whole brain and hippocampal volumes without causing adverse effects related to drug intake.

## 1. Introduction

Alzheimer’s disease (AD) is a condition with a progressive loss of problem-solving, language and memory and is the most common cause of dementia. Aging is one of the major risk factors for the late-onset, sporadic form of Alzheimer’s disease (SAD), which affects more than 90% of the subjects. The rare, familial form of AD (FAD) originally described by Alois Alzheimer [1] occurs in much younger subjects aged 30–50 and is characterized by the presence of extracellular plaques of beta amyloid (Aβ) and intracellular tangles of phosphorylated tau protein [2]. FAD was subsequently shown to be associated with mutations in several genes, including amyloid precursor protein, presenilin 1 and presenilin 2 [3].

There are many clinical, biomarker and pathological similarities between FAD and SAD. Carriers of the ApoE4 allele, which is an important risk factor for AD [4], develop more plaques than those expressing the ApoE3 allele [5]. However, the degree of cognitive impairment was found to be associated with the number of Aβ plaques in subjects with FAD but not in those with SAD, irrespective of the APOE allele. Moreover, Aβ plaques are also found in elderly subjects without dementia [6,7].

Further studies showed the relation between Aβ oxidative stress and phosphorylation of tau. An increase in phosph-p38 MAPK immunoreactivity was found at an early stage of the disease in the brains of subjects with AD. The p38 kinase enzyme resides quiescently in the cytosol until activated by dual phosphorylation on a specific domain (Thr^18o^ llu_Gly^181^ Tyr^182^) by stimuli that include H_2_O_2,_ TNFα and IL1β [8].

P38 is one of the enzymes that phosphorylates Tau, causing its aggregation [9]. Moreover, both p38 and tau phosphorylation can be induced in primary rat cortical neurons by incubation with the Aβ_(1–42)_ peptide, which causes oxidative stress by binding to heme and interfering with the respiratory chain in mitochondria, thereby increasing the production of H_2_O_2_ [10].

A wealth of data from human subjects and mouse models of memory loss induced by injection of human Aβ support its role in the etiology of dementia (reviewed by Selko and Hardy [11]). Thus, the role of Aβ in the etiology of AD has dominated the field of research and drug development for more than three decades. It has led the pharmaceutical industry to concentrate almost entirely on developing drugs to reduce the formation of Aβ or preparing monoclonal antibodies directed against insoluble Aβ in plaques [12] or its soluble protofibrils [13]. However, to date, only two of these eight Abs have resulted in any improvement in cognitive function, possibly because it is the soluble oligomers of Aβ in surviving neocortical synaptic terminals that cause accumulation of phosphorylated tau and is spread into the synapses, not that in plaques [14].

Thus, therapies designed to remove Aβ would be expected to be less effective once p-tau pathology has developed. Most of the Abs against Aβ also have unacceptable adverse effects, including MRI-detectable amyloid-related imaging abnormalities that may require hospitalization. Some subjects also experience headache, dizziness, difficulty in walking, confusion, vision changes and seizures [15]. The high cost of the only two antibodies approved by the FDA and the necessity to administer them intravenously make them unavailable to the majority to most subjects.

Therefore, it is time to focus on other morphological changes in the brains of patients with AD induced by soluble oligomers of Aβ, which have a much better correlation with the loss of cognitive function than the levels of Aβ.

## 2. Synaptic Plasticity

The synapse is the space formed by inter-neuronal connections into which different neurotransmitters are released, thereby enabling neurons to pass chemical signals to one another. Synapses are required for proper communication between neurons and can change their number, structure and function according to prevailing conditions. Synaptic plasticity is a process by which synaptic contacts are strengthened or weakened and is crucial for cognitive functions such as learning and memory (reviewed in Griffiths and Grant, 2023 [16]).

Abnormalities in synapses in the brains of subjects with AD were first described more than 55 years ago [17]. A later study showed that the significant loss of synapses in the frontal cortex in humans with AD was well correlated with the degree of cognitive deficit [18]. Synapse loss was found in the hippocampus of subjects with early AD and those with mild cognitive impairment (MCI) [19], which occurs several years before the onset of AD and is considered as a prodromal transition phase between normal aging and dementia. Patients with MCI experience a deficit in episodic memory and other subtle cognitive changes but no alterations in the activities of daily living [20]. To find new therapeutic targets for MCI and AD, it is critically important to understand what causes the loss of synaptic plasticity.

## 3. Mitochondrial Activity and Oxidative, Nitrative Stress

The brain consumes ~20% of the total body glucose, the majority of which is used to transduce energy through glycolysis and mitochondrial oxidative phosphorylation in order to support synaptic transmission [21]. Because of its lack of energy reserves, elevated metabolic activity and oxygen consumption, the brain is especially vulnerable to damage by oxidative stress. Brain glucose metabolism, measured by fluorodeoxyglucose-positron emission tomography (PET), was reduced in the brain of subjects with AD [22], those with MCI [23] and carriers of the apolipoprotein E epsilon-4 (ApoE4) allele, a major genetic risk factor for late-onset AD [4]. The age of onset was earlier in those with homozygotes with the ApoE4 allele than in heterozygotes [4]. This hypometabolism was not correlated with brain levels of Aβ [24,25].

Oxidative stress is a state of imbalance between the production of free radicals and their elimination by the organism’s anti-oxidative mechanism. Mitochondria are the main source of neuronal oxidative stress, since most free radicals are generated as byproducts of the mitochondrial electron transport chain. Aging reduces glucose availability and its neuronal uptake and the energy-transducing capacity of mitochondria, thereby increasing the likelihood of the formation of free oxygen radicals [26].

**Figure 1:**
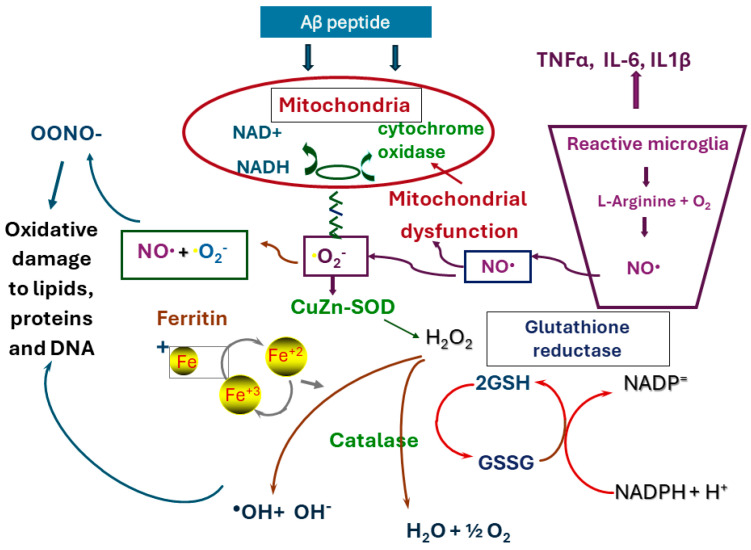
Interaction between mitochondrial dysfunction, oxidative stress and microglial activity. Cell damage caused by oxidative stress resulting from mitochondrial dysfunction activates microglia to produce nitric oxide (NO) and proinflammatory cytokines, thereby further exacerbating the damage. Red border compounds and enzymes within microglia; purple border reactions within microglia. CuZn-SOD = superoxide dismutase; NADH = nicotinamide adenine dinucleotide.

Reactive oxygen species (ROS) and reactive nitrogen species (RNS) are radical derivatives of oxygen and nitrogen, respectively. Nicotinamide adenine dinucleotide phosphate (NADPH) oxidase is the major source of the radical superoxide anion (O_2_•), which is converted into the hydrogen peroxide (H_2_O_2_) by superoxide dismutase and is the source of the highly reactive hydroxyl ion (OH•) [27]. NO and O_2_ interact to produce peroxynitrite, an extremely potent oxidant that causes damage to lipids, DNA, carbohydrates and proteins. Findings from several studies indicate that oxidative and/or nitrative damage of proteins [28,29] and lipids [30] occurs in the post-mortem brains of patients with AD, together with a deficiency in cytochrome c oxidase (complex IV) activity [31]. There are also signs of significant lipid oxidation [32] and nitrative damage [33] in the brains of subjects with MCI, confirming that these occur before the development of dementia. The hippocampus is one of the brain regions most severely affected by oxidative–nitrative damage in patients with AD. Memory loss results from its functional isolation from the entorhinal cortex and subiculum [33] (Figure 1).

ROS from mitochondria in presynaptic terminals promote the release of high levels of calcium from voltage-gated calcium channels that accelerate synaptic oxidative damage [34]. The crosstalk between Ca^2+^ and redox signals plays a key role in physiological functions in the brain. Thus, a high level of intracellular Ca^2+^ induces the stimulation of neuronal nitric oxide synthase, which leads to further production of ROS and RNS [35].

Soluble Aβ peptides localize within the mitochondrial membrane, interact with mitochondrial proteins and dysregulate the mitochondrial enzymes NADH dehydrogenase (Complex I) and cytochrome b and c1 (Complex III), thereby increasing the production of ROS [36]. Aβ also mediates dysregulation of calcium homeostasis and leakage of ions through the pores in the cell membrane [37].

## 4. Role of Microglia in Neuroinflammation

Microglia are the predominant, resident immune cells of the central nervous system (CNS). The use of two-photon microscopy in mice enabled the demonstration that microglia continuously survey the environment by means of their elongated processes [38]. This enables them to respond rapidly to alterations in brain homeostasis resulting from stress [39], trauma [40] and cell necrosis induced by ROS or RNS [41].

**Figure 2:**
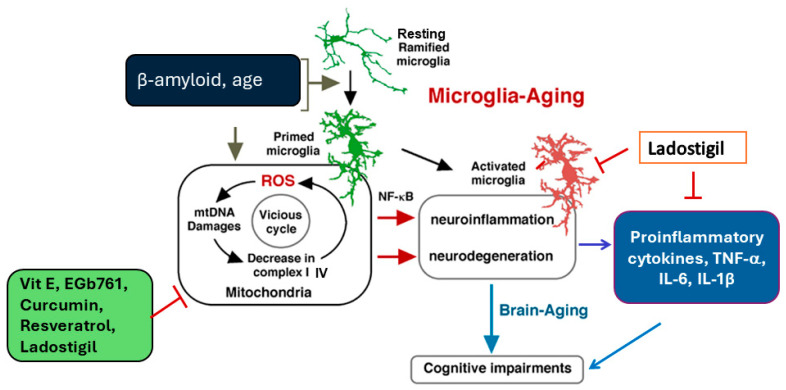
Role of microglia activation in neuroinflammation and its modulation by drugs. Arrows: activation; ┬ inhibition.

The microenvironment in grey and white matter brain regions shows considerable diversity in its metabolism and neurotransmitter profile [42]. Microglia also differ in their morphology in these regions. Those in grey matter have radially extending processes, while in white matter, they have a bipolar arborization [43]. After activation, microglia in grey matter have a higher expression of CD11b and CD45, retracted processes and an ameboid appearance [44]. After injury, or in the aging brain, microglia in white matter show thickened processes [45] (Figure 2).

Oxidative stress results in the activation of microglia and the release of pro-inflammatory cytokines. Microglia detect cell damage through their surface receptors, which respond to purines like adenosine triphosphate (ATP) and adenosine released from injured neurons [46]. Activation by ATP of the P2X type of purinergic receptor causes them to assume the reactive phenotype associated with phagocytosis: ameboid morphology with retracted branches and an enlarged cell body [47]. Stimulation of the P2X7 receptor subtype also triggers the efflux of K^+^ that activates the nucleotide-binding oligomerization domain-(NOD)-LRR and pyrin domain inflammasome (NLRP3) and converts procaspase-1 to caspase-1 [48]. This enables the processing and secretion of IL1β and other pro-inflammatory cytokines [49]. Adenosine activates Adora2a receptors (A2AR), which results in a loss of microglial processes, their retraction [50] and the release of Il-1β [51]. The expression of A2AR is significantly increased in the hippocampal neurons of aged humans and even more in those with AD [52,53,54].

Prolonged microglial reactivity and cytokine release results in a self-perpetuating state of chronic inflammation, thereby exacerbating tissue damage leading to neurodegeneration (reviewed in Green and Rowe [55]). Compared to those in young adults, microglia in aged rodents and humans have a lower threshold for activation [56] and a higher gene expression of inflammatory markers of the major histocompatibility complex (MHC class II) immune response pathway and pro-inflammatory cytokines like TNF-α and IL-1β [57]. IL-1β activates mitogen-activated protein kinase (MAPK) p38 and increases nuclear factor kappa-light-chain-enhancer of activated B cells (NF-κB) [58]. NF-κB is increased in the brains of subjects with neurodegenerative diseases [59].

## 5. Genes of the Immune System as Risk or Protective Factors in AD

Several genes that affect the immune system are either risk (S100B) or protective factors (Sirt3) for AD. They include S100B, an astrocytic protein with cytokine-like functions that is an accepted marker of glial injury. When released from astrocytes in response to injury, S100B contributes to disease progression by activating microglia and causing synaptic dysfunction and phagocytosis of neurons [60]. An increase in brain levels of S100B has been reported in Alzheimer’s disease [61].

TREM2 is a membrane-bound triggering receptor expressed on myeloid cells. The activity of TREM2 can be measured by evaluating the levels of its soluble fragment (sTREM2), which is generated during the TREM2 cleavage process. The concentration of sTREM2 in cerebrospinal fluid (CSF) is elevated in subjects with AD [62].

Mitochondrial deacetylase sirtuin-3 (SIRT3) is localized in the inner membrane and matrix of mitochondria, in which it acts as a sensor of mitochondrial energy. It is regulated by the metabolic co-factor (NAD+). SIRT3 controls a range of mitochondrial functions, including energy metabolism and protection against oxidative stress [63]. SIRT3 protects against age-related cellular dysfunction [64] and is the only sirtuin that is known to affect human lifespan [65].

Mutations in the PSEN1 gene encoding presenilin-1(PS1) are the most common cause of FAD. PS1 acts as the catalytic subunit of γ-secretase that cleaves the amyloid precursor protein (APP). PSEN1 mutations were shown to increase the Aβ_42_/Aβ_40_ ratio by lowering levels of Aβ_40_ [66].

The apolipoprotein E4 (*APOE4*) allele is a major risk factor for AD [67] and is also the strongest genetic risk factor for SAD, probably because of its role in lipid metabolism and related inflammation. APOE interacts with TREM2 and alters its inflammatory activity in response to injury [68,69].

## 6. Biomarkers for Aβ and Tau in AD

Aβ appears in the brain several years before the clinical symptoms of AD [70]. Therefore, Aβ biomarkers are crucial for early detection and intervention, when potential treatments might be more effective. One of the possible reasons for the failure of treatment to make an impact on AD is the lack of biomarkers that are readily measurable in clinical trials. PET scans for Aβ are time-consuming and expensive and not accurate enough to use in clinical trials. An ideal AD biomarker would identify key neuropathological characteristics, have a diagnostic sensitivity for AD over 80% and a specificity for differentiating AD from other dementias and be based on imaging or fluid biomarkers. PET for Aβ is clinically approved as a diagnostic test but is predominantly used for research. Cerebro-spinal fluid (CSF) or plasma biomarkers enable indirect detection of these pathologies. The concentration of Aβ_42_ in CSF and Aβ_42_/Aβ_40_ ratios correlates inversely with the cerebral amyloid-β plaque burden. The concentrations of total and phosphorylated tau (p-tau) correlate with the intensity of neurodegeneration and neurofibrillary-tangle pathology. Thus, a combination of CSF Aβ_42_ and p-tau181 increases sensitivity and specificity for distinguishing AD from non-AD pathologies to ~90% [71].

## 7. TREM2 in Combination with Aβ and Tau as Biomarkers for AD

In subjects with SAD, CSF levels of sTREM2 were significantly associated with those of total-tau and P-tau but not with Aβ42 [72]. Another study was performed in subjects with MCI and AD with a strong dependence on tau pathology and neurodegeneration or on amyloid-β deposition. A strong correlation was found between CSF sTREM2 levels and those of the inflammatory proteins TNF-α, TNFR1, TNFR2, ICAM1 and VCAM1, showing that it acts as a marker of neuroinflammation across the spectrum of early clinical AD [73].

## 8. Drugs That Reduce Oxidative Stress

Many natural and synthetic antioxidants and radical scavengers were claimed to prevent oxidative stress after they were assessed in cell systems and in animal models of AD (for detailed reviews, see [74,75,76,77,78]). Due to their poor intestinal absorption and brain penetrability, relatively few of them have been evaluated in placebo-controlled trials in subjects with AD or MCI. The findings from only those compounds are described below.

### 8.1. Vitamin E

Vitamin E is a generic term for a group of naturally occurring tocopherol and tocotrienol derivatives with biologic activity like that of α-tocopherol. These derivatives were shown to have antioxidant activity in a number of cell systems and animal models of oxidative stress. The plasma levels of α-tocopherol were found to be lower in subjects with AD and MCI than in healthy elderly individuals (reviewed in Grundman [78]). Some, but not all, retrospective studies showed a reduced risk of AD in those taking vitamin E supplements [79].

Unlike the evaluation of new drugs in placebo-controlled trials in patients with AD, natural substances like vitamin E were compared to other drugs, like donepezil (an acetylcholinesterase inhibitor) or selegiline (a monoamine oxidase B inhibitor), or a combination of them. One such study showed that both selegiline and vitamin E slowed progression of memory loss in subjects with AD [80]. However, in a placebo-controlled trial of 2000 IU/day of vitamin E, daily for 3 years in 769 subjects with the amnestic MCI, no significant difference was found in the rate of conversion to AD [81]. The lack of a clear effect in these studies may have been due to the source or nature of the tocopherol or tocotrienol derivative given.

### 8.2. Curcumin

Curcumin (1,7-bis(4-hydroxy-3-methoxyphenyl)-1,6-heptadiene-3,5-dione) is the main natural polyphenol found in the rhizome of Curcuma longa (turmeric) [82]. Turmeric has been known for thousands of years for its medicinal properties and has an extensive history as a medicinal herb in India [83]. This may explain why the prevalence of AD in patients between 70 and 79 years of age is 4.4-fold less than that of the United States [84]. At a concentration of 5 µM, curcumin can scavenge ROS and RNS [85] and restore levels of superoxide dismutase, catalase and glutathione peroxidase in macrophages exposed to H_2_O_2_, but higher concentrations are toxic [86]. In 19-month-old rats infused with Aβ_40_ and Aβ_42_ to induce neurodegeneration, curcumin given in the diet at a dose of (5.43 μmol/g) decreased the loss in synaptophysin and post-synaptic density 95 (PSD-95) in the cerebral cortex and improved the rats’ performance in the Morris water maze [87]. However, in a clinical trial in subjects with mild to moderate AD given 2 or 4 gm of curcumin daily for 24 weeks, no difference from placebo was found in several measures of cognition or behavior. The lack of effect of curcumin may have been due to its poor bioavailability after oral administration in humans, the short duration of the trial or both [88]. It is also possible that there are other active substances in turmeric which aid in the absorption of curcumin, which could explain the positive findings in India.

### 8.3. Resveratrol

Resveratrol (3,5,4-trihydroxystilbene) is a polyphenol found in the roots and fruit of plants including red grapes and mulberries. It was shown to be effective against memory impairment in a number of rodent models (reviewed in [89] and to reduce Aβ plaques in a transgenic mouse model of AD [90]. In a phase 2 trial in subjects with mild to moderate AD, resveratrol, given orally in a daily dose of 0.5–1 gm, had no effect on plasma Aβ_42_, CSF Aβ_42_, CSF tau, CSF phospho-tau_181_, hippocampal volume, entorhinal cortex thickness or different measures of cognitive function and even significantly reduced whole brain volume [91]. The failure of resveratrol to have any effect may also have been due to its poor oral bioavailability [92]. While analogs of resveratrol have been synthesized to provide better bioavailability [93,94], none so far has reached clinical studies for AD or MCI.

### 8.4. Ginkgo Biloba

Ginkgo biloba is one of the oldest medicinal plants and was used for the treatment of asthma in China 5000 years ago [95]. It was introduced into medical practice in Europe in 1965 by Dr. Willmar Schwabe III. This resulted in the preparation of an extract of Ginkgo biloba leaves containing 6% terpenoids and flavonoid glycosides like quercetin, 5–7% terpene lactones and 2.8–3.4% ginkgolides A. Its composition was adjusted according to the specifications of the European Pharmacopoeia to contain 22–27% flavonoid glycosides, 5–7% terpene lactones (2.8–3.4% ginkgolides A, B and C and 2.6–3.2% bilobalide) and less than 5 parts per million ginkgolic acids [96]. Ginkgo biloba extract (EGb) was shown to protect brain neurons against H_2_O_2_-induced oxidative stress [97], prevent aging of mitochondria [98] and decrease damage to cerebellar granule cells produced by a combination of H_2_O_2_ and FeSO_4_ [99]. EGb 761 (100 mg/kg) given for three weeks to aged female mice significantly improved short-term memory and membrane fluidity but had no effect on long-term memory [100].

Since the introduction of EGb 761 to medical practice in Europe, there have been many attempts to use it for preventing or reducing the severity of dementia [101,102]. A retrospective cohort study suggested that chronic administration of EGb 761 to subjects with MCI reduces the incidence of dementia [103,104]. Earlier, clinical trials indicated that EGb 761 was safe [101], but the clinical outcome in such trials was less clear, probably because of their short duration (12–52 weeks) and because real placebo treatment was not always given. In a trial in subjects with MCI aged 70 and more, EGb761 (240 mg/day) was given for a period of 5 years, while a control group was untreated [105]. About 5% of those in each group dropped out during this period, leaving 1228 subjects on EGb761 and 1259 controls. No significant difference was found in the number of subjects in each group that converted to AD; neither was there a difference in the number and nature of adverse events. Since the rate of conversion from MCI was much lower than expected, it is possible that the subjects were too healthy to have enabled detection of an effect of EGb761. On the other hand, in a trial by Ihl et al. [102] in subjects with AD, vascular dementia or both, EGb 761 240 mg/day given for 24 weeks produced a clinically significant improvement in cognition in 32% of 198 subjects with dementia compared to 15% of 200 subjects who received placebo. There was also a significant improvement of >4 points in the Neuropsychiatric Index total score in 45% of patients treated with EGb 761 and in only 24% of those taking placebo. The data from these compounds is summarized in Table 1.

## 9. Anti-Inflammatory Drugs

### 9.1. Non-Steroidal Anti-Inflammatory Agents

In 1990, McGeer and his colleagues made the original observation that subjects treated for several years with non-steroidal anti-inflammatory drugs (NSAIDs) for rheumatoid arthritis had a lower incidence of AD than untreated, age-matched controls. This was confirmed in several epidemiological studies [110,111]. Because NSAIDs are known drugs, such clinical trials are not readily funded and were only performed for 6–12 months in a small number of subjects [112]. A meta-analysis of clinical trials with several new NSAIDS in pain-free subjects with AD failed to find any association between their use and cognitive decline, with the exception of diclofenac [113,114]. The others may have been given at too late a stage of disease development.

### 9.2. Antibodies Against TNFα

Antibodies against TNFα, like eternacept, infliximab and adalimumab, were designed to treat rheumatoid arthritis, ulcerative colitis and Crohn’s disease [115,116]. They produce a significant improvement in these conditions. It was claimed that they were also likely to reduce the development of cognitive impairment in some older subjects [117]. This was based on the finding that perispinal injection of eternacept in a subject with AD resulted in an immediate and sustained improvement in cognitive function [118]. However, when given parenterally, they do not penetrate the brain. Moreover, after chronic administration in patients with rheumatoid arthritis, TNFα antibodies increase infections and reactivate latent tuberculosis, in addition to causing serious allergic reactions, lymphomas, congestive heart failure and demyelinating disease [119]. These effects probably result from complete blockade of the essential protective effects of TNFα.

Therefore, there is a real need for a drug that can prevent processes leading to mitochondrial dysfunction and oxidative stress and reduce excessive release of pro-inflammatory cytokines without blocking their essential activities. It should also be well absorbed from the gastro-intestinal tract after oral administration and readily penetrate the CNS.

## 10. Drug with Antioxidant and Anti-Inflammatory Activity

### Ladostigil (SPE100)

Ladostigil, 6-(N- ethyl, N- methyl carbamyloxy)-N propargyl-1(R)-aminoindan hemitartrate, was originally designed as an acetylcholinesterase (AChE) and monoamine oxidase (MAO)-B inhibitor to treat subjects with AD and depression [120]. However, its development for this purpose was discontinued because the addition of MAO-B inhibition conferred no advantage over other AChE inhibitors like rivastigmine or donepezil. Surprisingly, at 20–50-fold lower concentrations than those inhibiting either enzyme in vitro [120], ladostigil was shown to prevent the fall in the mitochondrial potential resulting from oxidative stress in SH-SY5Y neuroblastoma cells by delaying the opening of voltage-dependent anion channels [121].

In primary cultures of mouse microglia activated by a combination of lipopolysaccharide (LPS) and 2′-3′-O-(4-benzoyl benzoyl) adenosine 5′-triphosphate (BzATP), an agonist of P2X7 receptors, ladostigil significantly reduced the secretion of TNFα, Il-6 and Il-1β proteins at concentrations of only 10^−13^–10^−10^ M [122]. The combination of LPS and BzATP significantly upregulated the transcription factor early growth response 1 (Egr1). The severity of AD in human subjects correlates with the level of Egr1 [123]. Ladostigil decreased the gene expression of Egr1 and the nuclear translocation of EGR1 protein, while increasing that of TNFaIP3 (A20) and its protein levels in the microglial cytoplasm [122].

The nuclear factor kappa-light-chain-enhancer of activated B cells (NF-κB) is strongly associated with age in mice [123] and is increased in the brains of subjects with neurodegenerative diseases [124]. TNFaIP3 prevents the upregulation of NLRP3 inflammasome by NF-κB and conversion of pro-IL1β to mature IL-1β in activated macrophages [125]. Thus, by its action on these two key proteins, EGR1 and TNFaIP3, also in microglia, ladostigil decreases the release of pro-inflammatory cytokines from microglia.

Rats and other animal species lose their ability to discriminate between novel and familiar objects with advancing age [126]. Object recognition (OR) memory in rats bears some resemblance to short-term memory in humans [127]. While OR of 16-month-old Wistar rats was similar to that of 6-month-old adults, it was completely lost by 22 months of age. Chronic, oral treatment of the rats with ladostigil at a dose of 1 mg/kg/day for six months, from the age of sixteen months, restored their OR and spatial memory to that of young adult rats [128]. Ladostigil normalized microglial morphology in grey and white matter. This was significantly correlated with alterations in spatial memory in a specific manner in each brain area. Furthermore, the concentration of ladostigil in the plasma of the rats was compatible with that which caused maximal reduction in cytokine release from activated microglia [122]. In another study, it was found that 22-month-old rats had a significantly higher gene expression of A2AR and Cacna2d2 calcium channels in the hippocampus [129]. These proteins are associated with a loss of synaptic plasticity and are elevated in the hippocampus of aged humans [54]. In the parietal cortex, the rats had a higher expression of genes in activated microglia that encode proteins promoting the formation of pro-inflammatory cytokines (IL6, IL-1β, TNF-α), TNF-α receptor SF6 and IL-1 receptor type 1.

After six months of ladostigil treatment, the elevated gene expression of A2AR and Cacna2d2 calcium channels in the hippocampus was downregulated together with those associated with reactive microglia in the parietal cortex [129]. These effects of ladostigil could explain how it prevents the loss of OR and spatial memory in aging rats.

A phase 2 placebo-controlled trial of ladostigil hemitartrate given at a daily dose of 10 mg was initiated in 2015 in subjects with MCI [130]. The only end point then accepted by the European Regulatory Authority was the percentage of subjects who converted to AD. Since previous studies [131] had indicated that 15% of patients with MCI would progress to dementia each year, 100 were included in each drug and placebo group. However, it was found that the yearly conversion rate in the placebo group was only 7%. This indicated that many of them were probably too healthy to have been included in the study. Also, since 2011, the older population has become more aware of the importance of using their mental skills and to seek treatment of concomitant conditions like diabetes [132] and hypertension [133] that aggravate AD pathology. Another shortcoming in the trial was that about 30% in each group discontinued the study. Thus, only ~70 subjects remained in each group who had sufficient memory impairment to enable the detection of a difference in conversion rate. Nevertheless, ladostigil significantly reduced the percentage of converters among the ApoE4 non-carriers (*p* = 0.028). The average age of the nine ApoE4 carriers who converted to AD in the ladostigil group was almost 6 years more than that of the nine on placebo (*p* < 0.005). Those receiving ladostigil also had significantly lower mini-mental scores and more hippocampal atrophy, indicating that their cognitive function was probably too impaired when they entered the trial to have been affected by the treatment.

Other drugs evaluated in subjects with MCI or AD, like β or γ secretase inhibitors [134], antibodies against β-amyloid [135,136] or resveratrol [91], were all shown to reduce whole brain volume. By contrast, subjects given ladostigil showed a significantly slower rate of decline in whole brain and hippocampal volumes, suggesting a neuroprotective effect. In age-matched subjects in the study, ladostigil also slowed the rate of decline in delayed recall in the Rey’s verbal learning test [130]. Finally, none of the adverse effects observed during the three years of drug administration were considered different from those in the placebo group. A phase 3 study should include more accurate measures of magnetic resonance imaging in relevant brain regions, biomarkers of inflammation and oxidative stress and additional memory tests more appropriate for subjects with MCI.

## 11. Conclusions

To make a significant impact on AD, drug treatment must begin with MCI at the earliest signs of memory impairment. When designing a drug, prominence should be given to its effect on key processes that are responsible for neurodegeneration. These include impaired mitochondrial function, a common age-related process with consequent production of ROS and RNS, prolonged brain microglial reactivity and disruption of cortical and hippocampal synaptic plasticity. Reduction in synaptic plasticity shows a much better correlation with memory impairment for the common sporadic form of AD than the number of amyloid plaques or its soluble oligomers. Also, the drug should be sufficiently lipophilic to be absorbed from the intestine and readily penetrate the CNS. It should reduce the excess release of pro-inflammatory cytokines but not block their essential effects in the organism.

## Figures and Tables

**Table 1:** Effect of drugs with antioxidant activity.

Compound	Activity in Cells	Activity in Animal Models	Clinical Trials
Vitamin E (α tocopherol)	Vit E reduced cell death induced by H_2_O_2_ in hippocampal neuronal cells and by Aβ in rat hippocampal cell cultures [77]	Vit E improved cognitive performance in aged animals; prevented oxidative damage in animal models of AD [77]	Vit E (2000 IU/qd) given for 3 years slowed progression of memory loss in subjects with AD [80]. In patients with MCI, giving 2000 IU/qd produced no difference from placebo in rate of conversion to AD [81]
Curcumin (1,7-bis(4-hydroxy-3-methoxyphenyl)-1,6-heptadiene-3,5-dione) (active principle of turmeric)	Restored levels of SOD, catalase and glutathione peroxidase in macrophages exposed to H_2_O_2_ [85,86]	Curcumin (5.43 μmol/g) given in diet improved spatial memory of aging rats infused with Aβ_40_ and Aβ_42_ [87]	Curcumin, 2–4 gm qd given for 24 weeks to patients with mild to moderate AD, showed no difference from placebo in measures of cognitive function [88]
Resveratrol (3,5,4-trihydroxystilbene)	Resveratrol, 10–100 µM, reduced oxidative stress in human erythrocytes [106]	Resveratrol (300 mg/kg) reduced oxidative stress and number of plaques in Tg19959 transgenic mouse model [107]	Resveratrol (0.5–1 gm qd) had no effect on cognitive function, hippocampal volume or Aβ_42_, CSF Aβ_42_, CSF tau, CSF phospho-tau_181_ in patients with mild to moderate AD [91]
Ginkgo biloba extract (EGb761)	EGb761 (100 µg/mL) protected SH-SY5Y against H_2_O_2_-induced cell death and fall in mitochondrial function induced by Aβ (_1–42_) [97,98]	EGb761 (100 mg/kg) improved short-term memory and membrane fluidity avoidance learning in aged female mice [99]	EGb761 (240 qd) given for 5 years to subjects with MCI showed no significant difference from controls in % of converters to AD [105]. Subjects with dementia given EGb761 (240 qd) for 24 weeks showed a greater improvement in cognition than those on placebo [102]
Ladostigil (6-(N- ethyl, N- methyl carbamyloxy)-N propargyl-1(R)-aminoindan hemitartrate	Ladostigil prevented oxidative stress in SH-SY5Y neuroblastoma cells induced by H_2_O_2_ [108] and restored normal gene expression of NADPH and catalase	Ladostigil 1 mg/kg/day) restored object recognition and spatial memory in aging rats to those of young adults [109]	Ladostigil (1 mg/day) significantly slowed decline in hippocampal volume in patients with MCI and reduced number of subjects without ApoE4 gene who converted to AD
